# Implementation and Outcomes of a Telehealth Neonatology Program in a Single Healthcare System

**DOI:** 10.3389/fped.2021.648536

**Published:** 2021-04-23

**Authors:** Lory J. Maddox, Jordan Albritton, Janice Morse, Gwen Latendresse, Paula Meek, Stephen Minton

**Affiliations:** ^1^Intermountain Connect, Intermountain Healthcare, Salt Lake City, UT, United States; ^2^College of Nursing, University of Utah, Salt Lake City, UT, United States; ^3^RTI International, Research Triangle Park, Durham, NC, United States; ^4^University of Alberta, Edmonton, AB, Canada; ^5^Neonatal TeleHealth Intermountain Healthcare, Salt Lake City, UT, United States

**Keywords:** telehealth, newborn, resuscitation, implementation, telemedicine, transfers, length of stay, video-assisted resuscitation

## Abstract

**Background:** Intermountain Healthcare, an early adopter and champion for newborn video-assisted resuscitation (VAR), identified a reduction in facility-level transfers and an estimated savings of $1. 2 million in potentially avoided transfers in a 2018 study. This study was conducted to increase understanding of VAR at the individual, newborn level.

**Study Aim:** To compare transfers to a newborn intensive care unit (NICU), length of stay (LOS), and days of life on oxygen between newborns managed by neonatal VAR and those receiving standard care (SC).

**Methods:** This retrospective, nonequivalent group study includes infants born in an Intermountain hospital between 2013 and 2017, 34 weeks gestation or greater, and requiring oxygen support in the first 15 minutes of life. Data came from billing and clinical records from Intermountain's enterprise data warehouse and chart reviews. We used logistic regression to estimate neonatal VAR's impact on transfers. Negative binomial regression estimated the impact on LOS and days of life on supplemental oxygen.

**Results:** The VAR intervention was used in 46.2 percent of post-implementation cases and is associated with (1) a 12 percentage points reduction in the transfer rate, *p* = 0.02, (2) a reduction in spoke hospital (SH) LOS of 8.33 h (*p* < 0.01) for all transfers; (3) a reduction in SH LOS of 2.21 h (*p* < 0.01) for newborns transferred within 24 h; (4) a reduction in SH LOS of 17.85 h (*p* = 0.06) among non-transferred newborns; (5) a reduction in days of life on supplemental oxygen of 1.4 days (*p* = 0.08) among all transferred newborns, and (6) a reduction in days of life on supplemental oxygen of 0.41 days (*p* = 0.04) among non-transferred newborns.

**Conclusion:** This study provides evidence that neonatal VAR improves care quality and increases local hospitals' capabilities to keep patients close to home. There is an ongoing demand for support to rural and community hospitals for urgent newborn resuscitations, and complex, mandatory NICU transfers. Efforts may be necessary to encourage neonatal VAR since the intervention was only used in 46.2 percent of this study's potential cases. Additional work is needed to understand the short- and long-term impacts of Neonatal VAR on health outcomes.

## Introduction

Over 40 years ago, video technology was used to reduce newborn mortality and morbidity in high-risk maternal-newborn populations geographically separated from neonatologists (1). Over the past decade, the use and effectiveness of synchronous audio-video communications in pediatric care, newborn care, and support for newborn resuscitations has increased (2–4). Consumer demand, medical need, and federal reimbursement represent an acceptance of telemedicine and telehealth services (5–7). In the face of the overwhelming demand for telehealth services during the global pandemic, program evaluation becomes increasingly important despite the challenges of rapid cycle development, implementation, and success measures.

Telehealth video-assisted resuscitation (VAR) programs vary in implementation, and there is limited evidence of the impact of these programs. Three of the earliest VAR programs began in 2013. Randall Children's Hospital supported five low-risk maternity centers and participated in about two percent of all births (8, 9). The Mayo clinic also began using telehealth technology to support six spoke sites (10). Intermountain Healthcare conducted its first neonatal video consult in 2013. By early 2016, it had deployed the neonatal video consult service to over 16 hospitals in the Intermountain West.

Early in the implementation, NICU hub neonatologists and spoke sites shared anecdotal stories of successful VAR, preventing transfers, and increasing confidence in their ability to conduct a newborn resuscitation. This study was informed from early implementation success stories, Intermountain and UC Davis studies on reduced transfer rates, and improved resuscitation quality reported by Randall Children's Hospital and the Mayo Clinic (9, 11–13). This study's primary aim was to determine the influence of a neonatologist VAR on transfers to a NICU, birth facility length of stay, This study's primary aim was to determine the influence of a neonatologist VAR on transfers to a newborn intensive care unit (NICU), birth facility length of stay, and days of life on supplemental oxygen.

## Materials and Methods

In 2013, Intermountain piloted an innovative program to provide neonatal VAR to remote hospitals in Southwest Utah 54 and 118 miles away from the hub neonatal intensive care unit (NICU) in St. George, Utah. Over the next 3 years, this program was expanded to four NICU hubs and 16 spoke sites in Utah and its immediate borders. The Utah neonatal VAR project developed technical solutions, assessed clinical feasibility, conducted the implementation, and evaluated operational and clinical solutions. Two individuals on this paper were part of the implementation team, LM as operations manager and SM as neonatal telemedicine medical director.

Telehealth systems often operate with a “hub and spoke” model. In the case of Intermountain's newborn VAR program, the neonatologist staffed tertiary NICUs as the hub providing care via telehealth to smaller regional or community hospitals, the spokes. The newborn and family receive in-person care at the spoke facility. A single NICU hub will support multiple spoke hospitals as part of their regionalized maternal-newborn care system (14). This manuscript will refer to the NICUs as hubs, and local nursery's as the spokes.

### Telehealth Equipment

Design considerations for the neonatal environment include the diversity of newborn warmers, incubator designs, and the limited space around a warmer–approximately 48 inches deep and 25 inches wide. No telehealth equipment could be permanently attached to newborn warmers since warmers are FDA-regulated devices and are frequently moved throughout nurseries and hospitals.

**Figure 1:**
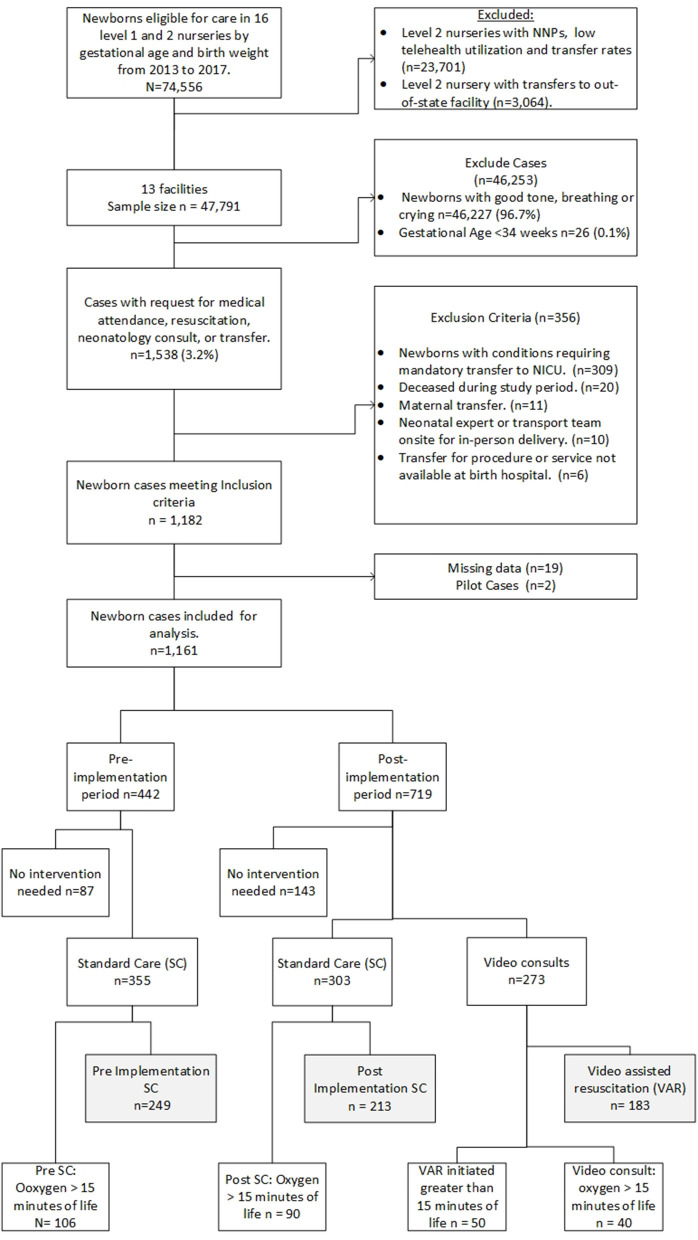
Sample collection.

Telehealth equipment was internally developed by the Intermountain telehealth technology team using currently available technology. The telehealth equipment included a palm-size Axis pan-tilt-zoom camera, a dedicated computer, and a monitor secured to the newborn area headwall or used as a mobile telehealth workstation (Figure 1). Microsoft video conferencing applications were customized to allow room selection, remote audio-video controls, and role-based access. Synchronous audio-video consults were conducted on Intermountain's intranet and approved by compliance and information systems security teams.

Each NICU hub had at least two telehealth-enabled workstations. Hardwired synchronous audio-video conferencing equipment was installed in delivery rooms, cesarean section operating rooms, and nursery locations in spoke hospitals. A mobile solution involved a palm-sized Axis camera, a Dell All-In-One computer, and Intermountain's customized video conferencing software. This allowed clinicians at spoke sites to access neonatologists for telehealth consults for at-risk neonates anywhere in their facility. Once the neonatologist was notified about the baby's location, the neonatologist could initiate a video connection, remotely control the camera using pan-tilt-zoom features, and adjust the audio for the spoke site hospital and themselves. These design features allowed the spoke site clinicians to focus on the newborn resuscitation, not the telehealth technology solution.

### Clinical Usability

Telehealth technologies were used to help spoke sites with simulation training, consults, and VAR, developing an early version of newborn resuscitation telemedicine program (NRTP) (5, 14–17). Clinical staff at spoke sites were asked to notify the neonatologists as early as possible, often before delivery, to allow time to establish a video connection. Indications for early notification for neonatologists were drawn from obstetrical high-risk categorization for mothers and fetuses (17, 18). Early notification provided time for neonatologists at the hub site to establish a video connection with the spoke site, discuss, and prepare clinical staff for the neonate's birth just as they would in an in-person delivery.

Systemwide implementation began after clinical feasibility, standardized telehealth equipment, and workflows had been established. At the end of 2016, there were 126 newborn telehealth in-room solutions and 14 mobile carts for newborn VAR; consults were being conducted at 16 spoke site locations. Hub site NICUs had at least two telehealth workstations to ensure that neonatologists had easy access to support emergent events.

## Study Design

In this retrospective, non-equivalent, pre-post telehealth implementation study, we analyzed a subset of newborns with no mandatory previously determined transport requirements. The study sample includes newborns with a gestational age of at least 34 weeks with oxygen administered within the first 15 min of life, born between 2013 and 2017. The newborns had a telephone or video neonatology consult or were transferred to a tertiary and quaternary NICU. Records obtained from the enterprise data warehouse (EDW) did not always include a scanned document indicating “resuscitation,” so manual chart review was completed. Chart reviews included minutes of life to oxygen administration, VAR or standard care, and days of life on supplemental oxygen.

Initiation of oxygen, resuscitation measures, or transfers were based upon medical necessity. These are clinical decisions not based on parental concern or ability to pay for services. Therefore, randomization to a control group is not possible. Intermountain Healthcare and the University of Utah Institutional Review Board approved this study.

### Data Collection and Analysis

**Figure 2:**
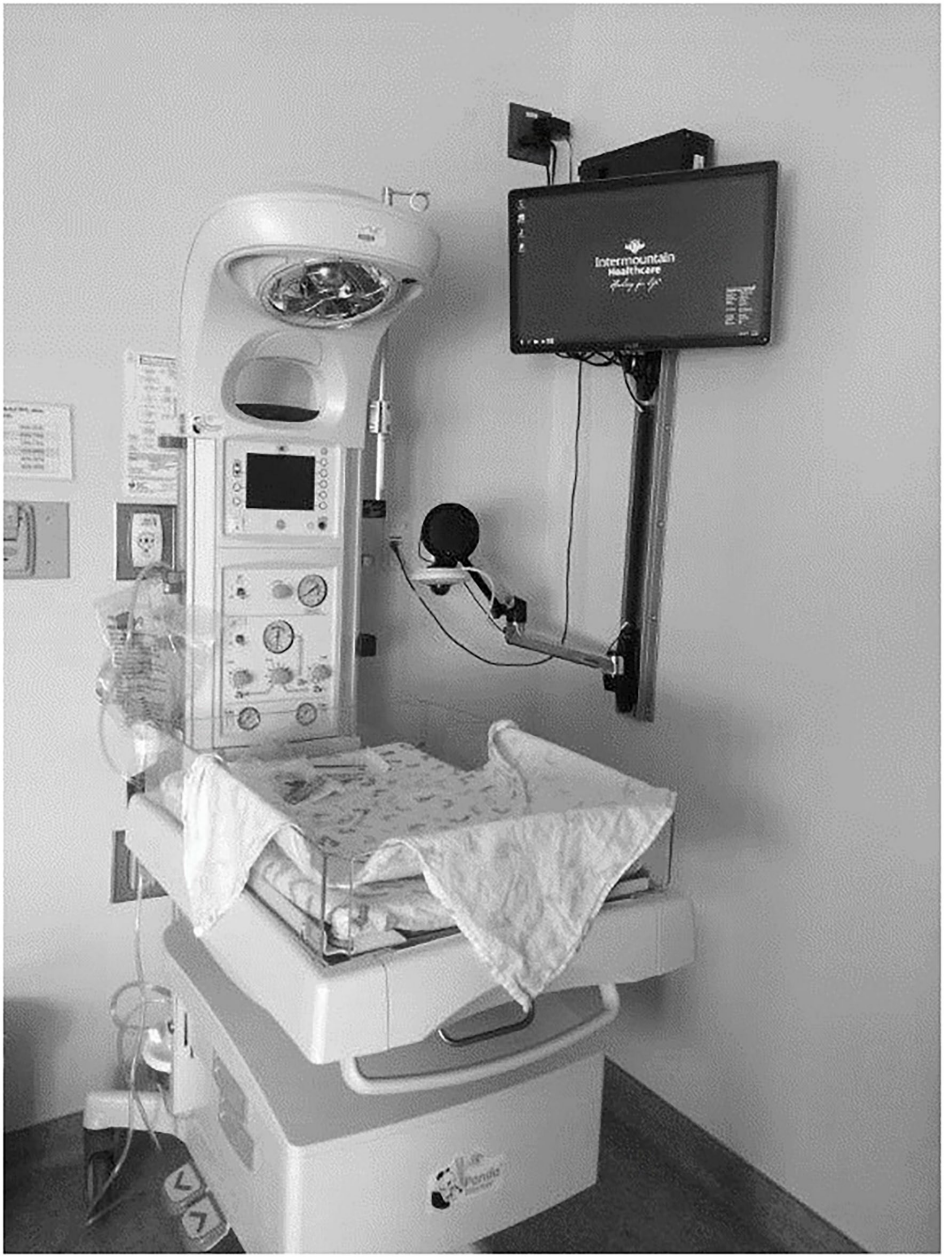
Wall mounted newborn telehealth station at spoke site.

Three groups were identified during data abstraction and initial analysis. Group 1 (*n* = 183) includes VAR conducted by the neonatologist after program implementation. Group 2 (*n* = 213) consisted of post-implementation standard care (SC) when neonatology consults were via telephone. Group 3 (*n* = 249), consisted of SC pre-implementation period. Two of the 16 spoke sites were excluded due to low telehealth and transfer rates. A third spoke site was excluded because newborns were transferred to an out-of-state NICU where follow-up chart review is not possible. Newborn diagnoses codes were used to exclude newborns with conditions requiring a mandatory transport to a quaternary or tertiary NICU. Additional exclusion criteria include newborns deceased during the study period, newborns transferred for maternal transports, newborns managed by in-person neonatology advanced practice practitioners, neonatologists, or transport team present at delivery, or mandatory transfers for service or procedure not available at the birthing hospital (Figure 2). Exclusion criteria were applied to generate a more homogenous population for this study. Additionally, we excluded cases (*n* = 50) where the VAR began more than 15 min after birth; in these cases, it was decided that it was unlikely that the neonatologist would have a significant impact on the resuscitation event. We conducted a sensitivity analysis to test the results and included cases with VAR >15 min of life.

All newborns were born in an Intermountain facility. Therefore, data from maternal and neonatal billing codes, diagnoses, clinical event data, and newborn gestational age, weight, gender, and Apgar scores were available to the researchers from the electronic medical record (EMR) and the EDW. Data on type of neonatology consult, minutes of life oxygen administered, and days of life on supplemental oxygen were abstracted from chart reviews, recorded in REDCap, and stored in a password-protected database. Consult types were determined from billing data and verified during chart review. Transfers and LOS data was determined from clinical events recorded in the EDW. Data discrepancies between billing data and chart review were reconciled after reviewing with the research team. A master data set combined all data sources.

### Statistical Analysis

We summarized characteristics for newborns with oxygen initiated in the first 15 min of life (*n* = 645) and the maternal and newborn diagnoses for the study population. All *t*-tests and *p* values are bivariate and presented for informational purposes only. There was no statistical difference in gestational age, weight, and gender for the newborns between the study groups. All newborns in this study had a 1-min Apgar score of <7. The mean 1-min Apgar score was 4.1 with a standard deviation of 2.4 in the VAR group and was significantly lower than the post-implementation SC group (mean = 5.0, SD 2.6), *p* < 0.001, and pre-implementation SC group (mean = 4.7, SD = 2.9) *p* = 0.03 (Table 1 Newborn sample characteristics and Table 2 Maternal and newborn diagnoses).

**Table 1:** Newborn sample characteristics.

	**Post-implementation**			**Pre-implementation**	
	**Video assisted resuscitation** **(*n* = 183)**	**Standard care** **(*n* = 213)**	***P***	**Standard care** **(*n* = 249)**	***P***
**Gestational age, mean (SD)**	38.2 (2.01)	38.1 (2.0)	0.58	38.4 (1.8)	0.40
**Gestational age category**, ***n*** **(%)^A^**			0.36		0.07
34 0/7–35 6/7 weeks	32 (17.5)	40 (15.2)		26 (10.4)	
36 0/7–37 6/7 weeks	39 (21.3)	73 (27.8)		75 (30.1)	
38 0/7–39 6/7 weeks	73 (39.9)	90 (34.2)		95 (38.2)	
40 weeks or greater	39 (21.3)	60 (22.8)		53 (21.3)	
**Birth weight (grams)**					
Mean (SD)	3,145 (599)	3,116 (587)	0.62	3,211 (510)	0.23
**Gender**			0.51		0.11
Female	62 (33.9)	97 (36.9)		103 (41.4)	
Male	121 (66.1)	166 (63.1)		146 (58.6)	
**APGAR, mean (SD)**					
1 min^B^	4.1 (2.4)	5.0 (2.6)	<0.001	4.7 (2.9)	0.03
5 min^B^	6.7 (1.8)	6.8 (1.9)	0.75	6.7 (2.2)	0.99
10 min^C^	7.4 (1.4)	7.4 (1.6)	0.64	7.3 (2.0)	0.85

*Results from t-tests with the assumption of unequal variances unless otherwise specified*.

A *Fishers exact test for categorical variables*.

B *VC, n = 180, Pre-UC, n = 246*.

C *VC, n = 74, Post-UC, n = 95, Pre-UC n = 132*.

**Table 2:** Maternal and newborn diagnoses.

	**Post-implementation**			**Pre-implementation**	
	**VAR**	**SC**	***P***	**SC**	***P***
	**(*n* = 183)**	**(*n* = 213)**		**(*n* = 249)**	
**Maternal diagnoses**, ***n*** **(%)**					
Chorioamnionitis	59 (32.2)	39 (18.3)	<0.001	58 (23.3)	0.04
Infection	24 (13.1)	26 (12.2)	0.95	9 (3.6)	<0.001
Hypertension, Pregnancy induced, Pre-eclampsia, eclampsia	45 (24.6)	30 (14.1)	<0.01	42 (16.9)	0.05
Mood and anxiety disorders	24 (13.1)	34 (16.0)	0.69	31 (12.5)	0.84
Diabetes mellitus (gestational, type 1 and 2)	17 (9.3)	30 (14.1)	0.56	29 (11.7)	0.05
Metabolic disorders other than diabetes	21 (11.5)	27 (12.7)	0.82	95 (38.2)	<0.001
Anemia or blood disorders	17 (9.3)	13 (6.1)	0.28	21 (8.4)	0.76
Obesity	8 (4.4)	18 (8.5)	0.15	17 (6.8)	0.27
Respiratory Disorders	6 (3.3)	12 (5.6)	0.49	20 (8.0)	0.03
Substance Abuse	5 (2.7)	9 (4.2)	0.30	5 (2.0)	0.63
Uterine bleeding, complications of labor	11 (6.0)	4 (1.9)	0.04	8 (3.2)	0.18
**Fetal diagnosis**, ***n*** **(%)**					
Multiple Gestation	27 (14.8)	20 (9.4)	0.11	8 (3.2)	<0.001
Small for dates	13 (7.1)	22 (10.3)	0.25	68 (27.3)	<0.001
Large for dates	14 (7.7)	15 (7.0)	0.82	6 (2.4)	0.09
Polyhydramnios	7 (3.8)	7 (3.3)	0.81	4 (1.6)	0.30
Oliogohydramnios	1 (0.6)	4 (1.9)	0.22	5 (2.0)	0.38
**Intrapartum diagnosis**, ***n*** **(%)**					
Abnormal fetal heart tracings	67 (36.6)	53 (24.9)	0.01	66 (26.5)	0.03
Abnormal presentation	57 (31.2)	46 (21.6)	0.03	42 (16.9)	0.00
Nuchal cord	50 (27.3)	44 (20.7)	0.12	54 (21.7)	0.18
Meconium associated with birth	40 (21.9)	28 (13.2)	0.02	43 (17.3)	0.24
Umbilical cord complications	18 (9.8)	17 (8.0)	0.52	59 (23.7)	0.00
Instrumental delivery	8 (4.4)	6 (2.8)	0.41	12 (4.8)	0.83
Placenta Previa, abruption, hemorrhage	16 (8.7)	8 (3.8)	0.04	16 (6.4)	0.38
General anesthesia	1 (0.6)	2 (0.9)	0.65	1 (0.4)	0.83
Narcotic use within four hours of delivery	1 (0.6)	3 (1.4)	0.38	3 (1.2)	0.46
**Newborn diagnosis**, ***n*** **(%)**					
Respiratory	146 (79.8)	166 (77.9)	0.65	181 (72.7)	0.09
Sepsis, actual, and rule-out	71 (38.8)	66 (31.0)	0.11	71 (28.5)	0.03
Fluid, electrolyte, and metabolic imbalances	58 (31.7)	59 (27.7)	0.39	63 (25.3)	0.15
Hypoxia	46 (25.1)	57 (26.8)	0.71	50 (20.1)	0.22
Pneumothorax	14 (7.7)	13 (6.1)	0.54	34 (13.7)	0.04
Cardiovascular disorders – other than congenital	17 (9.3)	18 (8.5)	0.77	36 (14.5)	0.09
Emphysema	–	2 (0.9)	0.15	34 (13.7)	<0.001
Abnormal movements, seizure assessment	6 (3.3)	9 (4.2)	0.62	31 (12.4)	0.001
Pneumonia	7 (3.8)	13 (6.1)	0.30	22 (8.8)	0.03
Hypoglycemia	18 (9.8)	24 (11.3)	0.64	20 (8.0)	0.52
Hypovolemia	22 (12.0)	17 (8.0)	0.19	14 (5.6)	0.02

We used logistic regression to identify factors associated with a neonatology consult (Table 3 Factors associated with neonatology consult before or within 15 min of birth). In this study, it was vital to parse neonatology VAR's effect, the independent variable, from the pre-and post-implementation period and confounding variables. Thus, in the main analyses, we controlled for factors significantly associated with early notification and other variables deemed important based on clinical expertise. Control variables included time (pre or post-implementation period, newborn gestational age, gender, multiple gestation, 1-min Apgar scores, maternal chorioamnionitis, pregnancy-induced hypertension, eclampsia, pre-eclampsia, hemorrhage, intrapartum abnormal fetal heart tones, newborn meconium, umbilical cord or placenta complications, and nursery level. All statistical tests are conducting with Microsoft ExcelPro 16.0 and Stata 15.1.

**Table 3:** Control factors for regression analyses.

	**AME^A^**	**SE^B^**	**95% CI**		***P***
			**LL**	**UL**	
**Post implementation (Time period)**	0.48	0.03	3.38	5.48	<0.001
**Maternal factors**					
Chorioamnionitis	0.11	0.04	0.29	1.29	<0.01
Infection	−0.03	0.33	−0.69	0.63	0.93
Hypertension, Pregnancy-induced, Pre-eclampsia, eclampsia	0.11	0.04	0.23	1.34	<0.01
Uterine bleeding, complications of labor	0.97	0.63	−0.25	2.20	0.12
**Fetal factors**					
Multiple gestation	0.11	0.05	0.10	1.46	0.02
**Intrapartum factors**					
Abnormal fetal heart tracing	0.05	0.03	−0.14	0.79	0.18
Meconium associated with birth	0.06	0.04	−0.17	0.99	0.17
Umbilical cord complications	0.06	0.05	−0.28	1.13	0.23
Placenta Previa, abruption, hemorrhage	0.09	0.06	−0.24	1.57	0.14
**Newborn factors**					
Gestational age	0.01	0.01	−0.08	0.18	0.47
Apgar, 1 min	−0.02	0.01	−0.24	−0.06	<0.01

*Number of observations = 639*.

A *Average marginal effects*.

B *Standard error*.

We used regression analyses to evaluate the impact of VAR on key outcomes. Because of the emphasis on the first few minutes, hours, and days of life, the data collected to evaluate clinical outcomes are not normally distributed. We used logistic regression to estimate the impact of VAR on transfer rates. Count variables were overdispersed, meaning the variance in the data is greater than the mean. Thus, we used negative linear regression to determine the neonatology VAR program's effects on newborn LOS and days of life on supplemental oxygen.

A pre-study statistical power analysis was performed for sample size estimation. The power analysis was based on data from an internal pilot study comparing the overall length of stay rates between pre-and post-implementation of the VAR program. The pilot study's effect size was 0.19, considered a small effect size, and was based on nursery level and newborns' gestational age but did not account for maternal or newborn risk factors. The sample size for this study was determined to be 565 cases with an alpha = 0.05, power = 0.80, a two-sided t-test.

## Results

In the logistic regression model, the VAR group had a significant decrease in the transfer rate of 12 percentage points, *p* = 0.02, SE = 0.05. For all transfers, the neonatology VAR intervention was associated with decreased LOS of 8.33 h, *p* < 0.001, SE = 1.3. For newborns remaining at the spoke facility, VAR was associated with a LOS reduction of 17.9 h, *p* = 0.06, SE = 9.5. For newborns transferred within 24 h, VAR was associated with a reduced LOS by 2.21 h, *p* < 0.01, SE = 0.60.

Neonatal VAR influenced days of life on supplemental oxygen. For newborns transferred to a NICU, neonatal VAR was associated with a reduction in days of life on supplemental oxygen by 1.41 days, *p* = 0.08, SE = 0.80. Newborns that were not transferred spent an average of 9.84 h less (0.41 days), *p* = 0.04, SE = 0.20, than the standard care groups.

(Table 4 VAR influence on transfers, birth facility length of stay, and days on supplemental oxygen).

**Table 4:** VAR influence on transfers, birth facility length of stay, and days on supplemental oxygen.

**Outcome metrics**	**Number of observations**	**AME**	**SE**	***P***
Percentage point reduction in transfers^*^	639	−0.12	0.05	0.02
Reduced LOS in hours for all transferred newborns	311	−8.33	1.33	<0.01
Reduced LOS in hours for newborns remaining at the birthing facility	328	−17.85	9.47	0.06
Reduced LOS in hours for newborns transferred within 24 h	273	−2.21	0.60	<0.01
Reduced days on supplemental oxygen, transferred	216	−1.41	0.80	0.08
Reduced days on supplemental oxygen, not transferred	303	−0.41	0.20	0.04

*Linear regression was used to assess the effect of the VAR intervention on these outcomes unless noted otherwise*.

*All models used the same control factors listed in Table 3*.

* *Logistic regression analysis was used to determine transfer rates*.

The sensitivity analysis, including 50 additional cases with VAR conducted later than 15-min of life, produced similar results to those just reported. The VAR group transfer rate increased to 14 percentage points, *p* < 0.01, SE = 0.04. For all transfers, the neonatology VAR intervention was associated with decreased LOS of 7.03 h, *p* < 0.001, SE = 1.24. For newborns remaining at the spoke facility, VAR was associated with a LOS reduction of 16.71 h, *p* = 0.07, SE = 9.05. For newborns transferred within 24 h, VAR was associated with a reduced LOS by 1.74 h, *p* < 0.01, SE = 0.58.

For newborns transferred to a NICU, neonatal VAR was associated with reduced days of life on supplemental oxygen by 1.39 days, *p* = 0.76, SE = 0.07. There was no change in days of life on supplemental oxygen for newborns remaining at the spoke site.

## Discussion

Intermountain Healthcare's neonatology service was an early adopter and champion for VAR. This program was implemented to provide expert support for high-risk births and post-delivery care to reduce unnecessary transfers (15). Guidelines to request additional medical assistance from an on-call pediatrician or another qualified medical provider were in place before the neonatology VAR program. During program implementation, spoke sites were encouraged to follow existing guidelines to request on-call medical attendance. Once the on-call provider was notified, the neonatologists would be called for an anticipated VAR. Frequently, the neonatology video consult would be established before the in-person medical provider's arrival. Establishing a video connection before birth allowed the neonatologist to receive a report, anticipate clinical scenarios, review resuscitation protocols, and emergency resuscitation equipment with the spoke site team (5, 15, 19).

In this study, acute maternal diagnoses of chorioamnionitis, pregnancy-induced hypertension, eclampsia, pre-eclampsia, hemorrhage, intrapartum abnormal fetal heart tones, and fetal meconium, umbilical cord, or placenta complications were most frequently associated with a request for a neonatology VAR before or within 15 min of birth. An acute maternal, intrapartum, or fetal event's urgency may explain the higher frequency of these diagnoses in the VAR group. Ideally, these mothers would be transferred before birth to a regional maternity center equipped to manage these high-risk patients. However, maternal transfers are not possible when mothers with these conditions present to community and rural hospitals in advanced labor. Neonatal VAR acts as a safety net for these high-risk newborns by providing similar standards of care as the NICU hub (15, 20). Chronic maternal conditions such as hypertension, diabetes, or mood and anxiety disorders were higher in the post-implementation, standard care group. Pediatricians and family practice clinicians may feel more comfortable managing these patients without neonatal expert support at birth.

### Outcomes

Transfers, spoke site LOS, and days on supplemental oxygen were used as outcome measures to evaluate this neonatal VAR program (19). Direct measurements of resuscitation quality were not available in the newborn record. We identified a relatively homogenous group of newborns and chose transfers, LOS, and days on supplemental oxygen outcomes as indirect measures of the influence of VAR on resuscitation quality. This study is one of the earliest to report LOS and days on supplemental oxygen for neonatal VAR interventions at spoke sites.

In this study, neonatal experts were called to assist with the most acute maternal, fetal, and newborn conditions. Although higher-risk newborns are represented in the VAR group, VAR is associated with fewer transfers and supports prior studies that telehealth consults are associated with reduced transfer rates (11, 13). Additionally, VAR newborns transferred within the first 24 h of life had a LOS reduction of 2.21 h. A benefit of telehealth is that patients can be more efficiently triaged to the appropriate level of care when specialists are involved with their care (15, 21).

Reductions in days of life on supplemental oxygen were also associated with VAR. Results from a simulation study using video consults for pre-transport evaluation found that neonatologists used less invasive respiratory support, i.e., continuous positive airway pressure (CPAP) vs. intubation, for transport (22). Neonatologists' tendency to use non-invasive ventilation techniques may contribute to fewer days on supplemental oxygen in the VAR group. Neonatology support for pre-transport stabilization and preference for CPAP vs. intubation for mild to moderate respiratory distress may also account for shorter LOS at spoke sites.

The sensitivity analysis we conducted suggests that neonatal VAR gains are the most beneficial when the VAR occurs at birth or within the first 15 min of life. When the neonatal expert is waiting for birth, they can receive a report and prepare bedside teams for a high-risk birth. The benefits of an early VAR intervention and the opportunity for “just in time” education offset the 20 percent of all medical attendance requests at birth that did not require NRP interventions.

The informal training spoke sites receive from ongoing communication and relationships with neonatal experts helps build knowledge and skills acquired during NRP certification. These ongoing synchronous audio-video interactions build upon existing telehealth-based simulation education and NRTP to improve clinical outcomes (11, 23–25). We were encouraged that our VAR rate was 46.2 percent, higher than the expected 34.5 percent rate reported by Fang et al. (26), reporting that 65.5 percent of users “did not use service because they did not have a clinical need.” This study was not designed to explain why the spoke site chose (1) not to contact a neontologist for a VAR, (2) delay the consult until a transfer was required, or (3) why the NICU hub and spoke sites used the telephone. These questions deserve future study, especially in the post COVID-19 period, when telehealth is the only plausible alternative to in-person care. The rapid adoption and implementation of telehealth during the COVID-19 pandemic and improved outcomes demonstrated in this study may persuade medical providers to increase telemedicine usage (27).

Paradoxically, the combination of increased neonatal video consults and steady transfer rates in the UC group led to an overall increase in neonatal consults in the post-implementation period. When establishing a neonatal VC program for newborn resuscitation and initial stabilization, there may be a period of increased neonatology workload. A telehealth service introduces new technology, workflows, and uncertain demand. Sample selection for this study provides a guideline for estimating demand for future neonatal video consult programs. Considerations for estimating the frequency of neonatal VC include:

1. Pre-transport stabilization cases for all premature and required newborn transfers.
2. Cases in which medical attendance at birth was requested or newborn resuscitation measures were performed.
3. Current transfer rates.

Estimates for neonatal video consult programs should allow for a period of technology deployment, testing, education, and early program adoption when both standard care transfers and VAR overlap.

### Limitations and Future Directions

This study's retrospective design limits this study with sample selection from a single healthcare system and newborns >34 weeks gestation requiring oxygen within the first 15 min of life. Differences in spoke sites were controlled by nursery level, not by the implementation date. Since implementation occurred over a 16-month timeline, early spoke sites had more time to use telehealth during this study period. We used regression modeling to control for confounding variables associated with non-randomized studies.

Due to technology limitations and retrospective chart review, it was not feasible to determine each video consult's length of time. We may be missing data in the pre-implementation period since neonatology consults were not always documented. In some instances, resuscitation events were reconstructed during chart review. VAR within 15 min of life was chosen as the cut-off period, with approximately 80 percent of all VAR occurring before or within the first 15 min of life. We recognize that there may not be a difference between a VAR at 14 or 16 min but had to establish the study population. Newborns with a VAR >15 min of life were not included in this study. However, they were included in the sensitivity analysis which produced similar results.

Randomized controlled trials are challenging in real-world clinical settings, especially when an intervention, VAR, reduces transfers, facilitates timely triage, reduces LOS, and reduces days on supplemental oxygen. When and where VAR is available, we must ensure equitable access to high-quality neonatology care regardless of geographical location (15). Future studies should include prospective, observational, and ethnographic studies that emphasize decision-making to activate neonatology support in the delivery room. Team building and communication skills are critical areas of decision-making and can be studied in a simulated or clinical setting. Telehealth fundamentally changes communication styles and perceptions when the specialist is visible to the entire spoke site team, parents, and loved ones in the delivery room. Video recording of newborn resuscitation events affords clinicians the opportunity for an objective review of their performance like an elite athletes' review of their performance and provides an opportunity for coaching. In addition to using outcomes for quality improvement, transfer, LOS, and days of life on supplemental oxygen can be quantified for payers, hospitals, patients, and communities to describe a comprehensive neonatal VAR and NRTP valuation.

## Conclusion

Improvements in care processes and outcomes provide evidence that neonatal VAR improves care quality. Neonatal VAR also helps increase the capabilities of local hospitals and keeps patients in their communities. There is an ongoing demand for support to rural and community hospitals for urgent newborn resuscitation, and complex, mandatory NICU transfers. Still, efforts may be necessary to encourage the use of neonatal VAR as the intervention was only used in 46.2 percent of potential cases in this study. Additional work is needed to understand the short- and long-term impacts of Neonatal VAR on health outcomes.

## Data Availability Statement

The raw data supporting the conclusions of this article will be made available by the authors, without undue reservation.

## Ethics Statement

The studies involving human participants were reviewed and approved by Intermountain Healthcare and the University of Utah. Written informed consent from the participants' legal guardian/next of kin was not required to participate in this study in accordance with the national legislation and the institutional requirements.

## Author Contributions

LM, JA, JM, and SM conceived this study. LM and JA obtained and analyzed the data and contributed equally to this work. GL, PM, and JM supervised this study and share senior authorship. SM is last author and medical director for this program. All authors read, edited, and approved the final manuscript.

## Conflict of Interest

The authors declare that the research was conducted in the absence of any commercial or financial relationships that could be construed as a potential conflict of interest.
